# Fostering Community Partnerships

**DOI:** 10.1093/geroni/igaf122.1288

**Published:** 2025-12-31

**Authors:** Steffi Kim

**Affiliations:** University of Alaska Anchorage, Anchorage, Alaska, United States

## Abstract

The importance of building local and national partnerships to address the crucial need to share culturally sensitive knowledge related to brain health, resources and support for dementia caregivers, and dementia knowledge in remote Alaska Native communities is well known. This presentation will discuss the partnership-building process and its impact on communities at the family, community, and state levels. Remote Alaska Native communities have unique needs in addressing health disparities, access to resources, and the latest health-related knowledge. With increasing numbers of Alaska Native people being diagnosed with dementia, it is crucial to continue to share information on brain health, warning signs of dementia, and what dementia is, as well as to develop resources that specifically speak to the needs of those living in these communities. Alaska Native culture has shown to be a factor that protects people caring for someone with dementia from stigma. Knowledgeable and responsive Alaska Native communities minimize the strain on caregivers and increase the ability of the Elder to age within the community. Through collaboration, the AN CARE Lab seeks to advance knowledge and engage community members in developing resources. Community partners such as the Alzheimer’s Resources of Alaska, the Commission on Aging, the Aleutian Pribilof Island Association, the Alzheimer’s Association Alaska Chapter, and the Alaska Department of Health are engaging with communities, supporting community projects, and participating in state-level advocacy to include the needs of Alaska Native community members.

